# A simple method for characterizing left ventricular remodeling by cardiovascular magnetic resonance

**DOI:** 10.1186/1532-429X-13-S1-P277

**Published:** 2011-02-02

**Authors:** Shawn C Pun, Maria Figura, Kelvin Chow, Mark Haykowsky, Richard Thompson, Ian Paterson

**Affiliations:** 1 University of British Columbia, Vancouver, BC, Canada; 2 University of Alberta, Edmonton, AB, Canada

## Background

Remodeling of the left ventricle (LV) occurs in response to various physiological and pathophysiological conditions. The American Society of Echocardiography recommend that LV remodeling be described as the relationship between LV mass and LV wall thickness based on 1-D and 2-D measurements. Cardiac magnetic resonance (CMR) provides accurate 3-D measures of LV volumes and mass; however, there is no universally agreed upon approach to measure remodeling. We propose using the ratio of LV mass to LV end-diastolic volume (LVEDV) as the left ventricular remodeling index (LVRI).

## Objectives

To describe and compare patterns of LV remodeling in various cardiac conditions using CMR. We hypothesized that the LVRI would accurately reflect underlying pathophysiology.

## Methods

A total of 105 consecutive cases (89 males, mean age 48±18), with elevated LV mass (referenced to Hudsmith et. al., JCMR 2005) from 2006-2009 were obtained from the CMR clinical database at our institution. 32 healthy volunteers served as controls. Based on the clinical history and the qualitative CMR findings, cases were categorized as: inflammatory (INF), dilated cardiomyopathy (DCM), ischemic cardiomyopathy (ICM), pressure loaded (PL) (eg. aortic stenosis and systemic hypertension) and volume loaded (VL) (eg. aortic regurgitation and mitral regurgitation).

Quantitative volumetric analyses were performed using standard imaging analysis software (Leonardo, Siemens). A short axis stack of steady state free precession cines were used to obtain LV mass and LVEDV by methods of disks. The LVRI was calculated from the ratio of LV mass to LVEDV. Statistical significance was assessed using an unpaired Student’s T-test.

## Results

The DCM, ICM and PL groups were significantly older and the INF group had a greater proportion of males compared to controls. There was no statistically significant difference in LVRI between males and females or between older (age >35) and younger adults.

The mean LVRI for controls was 0.87±0.1g/ml. Compared to controls, mean LVRI was elevated in INF (0.99±0.15g/ml, p=0.002) and PL (0.98±0.12g/ml p=0.002). Conversely, mean LVRI was reduced in ICM (0.79±0.13g/ml, p=0.014) and VL (0.74±0.13g/ml, p<0.001). There was no statistically significant difference in mean LVRI between DCM and controls. Table 1.

**Table 1:** 

	Controls	Inflammatory (INF)	Dilated Cardiomyopathy (DCM)	Ischemic Cardiomyopathy (ICM)	Pressure Loaded (PL)	Volume Loaded (VL)
Number	32	20	34	20	16	15
Age	39 ±13	35 ±16	49 ±15*	62 ±14*	51 ± 18*	45 ± 22
Male (%)	18 (56%)	18 (90%)*	26 (77%)	14 (70%)	11 (69%)	12 (80%)
BSA (m^2^)	1.96 ± 0.2	2.08 ±0.2	2.03 ±0.3	2.01 ±0.3	2.00 ±0.3	2.00 ±0.2
LVEF (%)	62 ±4	53 ±13*	38 ±18*	31 ±12*	62 ±11	51 ±11*
LVEDV Index (ml/m^2^)	80.5 ± 19	87.7 ±26	126.4 ± 42*	106.1 ± 24*	82.3 ± 16	150 ± 46*
Mass Index (g/m^2^)	69.2 ±13	84.4 ±17*	104 ±22*	137.1 ±34*	79.9 ±15*	108 ±26*
LVRI (g/ml)	0.87 ±0.1	0.99 ±0.15*	0.86 ±0.18	0.79 ±0.13*	0.98 ±0.12*	0.74 ±0.13*
LVRI (%) >2SD control	NA	5 (25%)	12 (35%)	3 (15%)	4 (25%)	5 (33%)

All values are presented as Mean±SD. *P-value <0.05 compared to healthy controls. Abbreviations: BSA (Body surface area), LVEDV (Left ventricular end diastolic volume), LVEDV Index (LVEDV/BSA), LVEF (Left ventricular ejection fraction), LVRI (Left ventricular remodeling index = LV Mass/LVEDV), LVRI (%) >2SD control (proportion of LVRI greater than 2SD above control mean), Mass Index (Mass/BSA).

## Conclusions

LVRI is a simple method for quantifying in a variety of cardiac conditions. The observed values reflect underlying pathophysiology, with elevated indices in myocardial edema or increased afterload, and decreased indices with LV wall infarction or increased preload. Future studies should assess its role in the serial assessment of ventricular geometry and at earlier stages of disease.

